# Future healthcare professionals’ knowledge about the Argentinean abortion law

**DOI:** 10.5116/ijme.56e0.74be

**Published:** 2016-03-28

**Authors:** Belén Provenzano-Castro, Silvia Oizerovich, Babill Stray-Pedersen

**Affiliations:** 1 Department of OB-GYN, Gynaecology Division, Faculty of Medicine, University of Buenos Aires, Argentina, Ciudad de Buenos Aires, Argentina; 2 Division of Gynaecology and Obstetrics, Institute of Clinical Medicine, University of Oslo, Norway

**Keywords:** Healthcare students, abortion law, knowledge, personal opinion, Argentina

## Abstract

**Objectives:**

We assessed healthcare students’ knowledge and opinions on Argentinian abortion law and identified differences between first- and final-year healthcare students.

**Methods:**

In this cross-sectional study, self-administered anonymous questionnaires were administered to 760 first- and 695 final-year students from different fields of study (medicine, midwifery, nursing, radiology, nutrition, speech therapy, and physiotherapy) of the School of Medicine at the University of Buenos Aires, in 2011-2013.

**Results:**

Compared to first-year students, a higher percentage of final-year students knew that abortion is legally restricted in Argentina (p < 0.001). A significantly higher percentage of final-year students could correctly identify the circumstances in which abortion is legal: woman´s life risk (87.4% last vs. 79.1% first year), rape of a woman with developmental disability (66.2% first vs. 85.4% last-year; p < 0.001). More final-year students chose severe foetal malformations (37.3% first year vs. 57.3% final year) despite its being illegal.

**Conclusions:**

Although most final-year students knew that abortion is legally restricted in Argentina, misconceptions regarding circumstances of legal abortion were observed; this may be due to the fact that abortion is inadequately covered in the medical curricula. Medical schools should ensure that sexual and reproductive health topics are an integral part of their curricula. Healthcare providers who are aware of the legality of abortion are more likely to provide the public with sound information and ensure abortions are appropriately performed.

## Introduction

In developing countries, reduction of maternal mortality is a health policy priority, due to large numbers of avoidable maternal deaths. One of the United Nations Millennium Development Goals (MDG 5) is to lower maternal mortality and achieve universal access to reproductive health.^1^^,^^2^

Abortions performed according to medical guidelines carry very low risk of complications; unsafe abortions contribute substantially to maternal morbidity and death worldwide, representing one of the major causes of preventable maternal deaths.^3^ World Health Organization data show that the global fatality rate associated with unsafe abortions is 700–1,000 times higher than that associated with legal induced abortions.^3^

In South America, three million induced abortions occurred yearly between 1995 and 2008; that is, 25% of all pregnancies ended in abortion. Almost all of them occurred under unsafe conditions.^4^

Since 1990, the International Conference on Population and Development and United Nations women’s conferences have advocated that whenever abortion is legal, it should be made accessible under safe conditions.^5^^-^^7^ In Latin America, where abortion is legally restricted in most countries, these recommendations have rarely been followed.^4^^,^^7^^, ^^8^

In Argentina, abortion is legally restricted to situations in which the woman’s life or health is in danger or if pregnancy is the result of rape or assault of a woman with developmental disabilities. However, the healthcare system does not guarantee access to legal abortion to most women who meet its criteria; they are either not informed of their rights or their access to the practice is impeded. This forces them to resort to unsafe abortions.

Annually, 370–460,000 abortions are estimated to be performed in Argentina, most of them under unsafe conditions. This has resulted in one third of the maternal deaths during the past decade.^9^^-^^13^ Moreover, in 2013, 50% of maternal deaths (25) due to abortion occurred among women aged 15–29 years; nine of them were adolescents.^14^^, ^^15^

High-quality sexual and reproductive healthcare services are key to improving adolescents’ and women’s health and to lower maternal mortality. Healthcare students are future healthcare providers. Medical doctors, midwives, and nurses play key roles in providing the public with adequate information, education, and access to sexual and reproductive health. Healthcare providers should encourage safe practices and facilitate access to contraception and abortion when legally permitted. Medical schools should include sexual and reproductive health education in their curricula to ensure better professional standards.

The School of Medicine of the University of Buenos Aires (UBA) is one of the largest in Argentina. Annually, there are around 5,000 students registered and 2,300 graduates. Our previous investigation among its first-year students revealed a notable lack of knowledge on current abortion regulations in Argentina. Moreover, those first-year students who were aware that abortion was legally restricted could not identify the circumstances in which it was permitted.^16^

The objective of this study is to compare knowledge and personal opinions on the abortion law between first and final-year healthcare students of the School of Medicine of the University of Buenos Aires. From this, we will infer what they have learned at school about the Argentinean abortion law.

## Methods

### Study design

A cross-sectional study was conducted from 2011 to 2013. Students from the School of Medicine of the UBA (majoring in medicine, midwifery, nursing, radiology, nutrition, speech therapy, and physiotherapy) completed an anonymous questionnaire on sexual and reproductive health.

Ethical approval was obtained from the Ethical Committee of the Facultad de Medicina, Universidad de Buenos Aires and The Regional Committee of Health Research Ethics (REK), Norway (2014/1244).

### Sampling

A first- and a final-year class majoring in the fields of study surveyed (medicine, midwifery, nursing, radiology, nutrition, speech therapy, and physiotherapy) were selected to answer the questionnaire, using multistage random probability sampling. Non-probability sampling was employed to select participants. Only those students present on the day of the survey answered the questionnaire.

### Data collection methods

We designed a self-administered multiple-choice questionnaire after reviewing similar surveys from Argentina and other countries.^17^^-^^20^ Since this investigation was part of a larger study, the questions asked about background information and knowledge on sexual health, contraception, sexually transmitted infections, abortion, and legislation. In addition, questions about personal experiences and opinions were included.

One question asked about the status of abortion in Argentina; response options were ‘legal’, ‘legally restricted’, ‘illegal’, or ‘do not know’. If they responded with the option ‘legally restricted’, they were asked to identify the legally permitted options from a list of possible circumstances.

Following these responses, students were asked for their opinions on how abortion should be legally treated in the country: ‘legal’, ‘legally restricted’, ‘illegal’, or ‘do not know’. Those who answered that it should be ‘legally restricted’ were asked to select conditions under which it should be legal from a list of possible circumstances.

Before the main study, the questionnaire was pilot-tested among a small group of students and underwent minor revisions.

### Procedure

Arrangements were made with faculty members and lecturers to allot dates to administer the questionnaire.

Students were given an explanation of the study’s purpose and intent; then they provided informed consent. Participation was voluntary and the information collected was confidential and anonymous. No incentives were offered for participation. The researchers remained present while students completed the questionnaire in case doubts or questions arose. The questionnaire took about 30 minutes to complete.

### Data analysis

The sample was weighted for gender and major using UBA School of Medicine statistics.

Frequencies and cross-tabulations were calculated. Univariate and bivariate analyses were performed. Chi-square, Fisher, and other tests as appropriate were conducted to compare first- and final-year students’ responses. IBM SPSS v. 20 and Epidat 3.1 were used for the analysis. The significance threshold was 0.05.

Students who majored in radiology, nutrition, speech therapy, and physiotherapy were grouped as ‘other studies’ or ‘controls’ because they did not have sexual and reproductive health education in their curricula. Their answers were compared with those given by medical, midwifery, and nursing students.

## Results

The questionnaire was completed by 1,489 healthcare students (781 first-year students and 708 final-year students). Students who did not provide their nationality, major or field of study, or birth year were excluded, leaving a total of 760 and 695 respondents from first and final years respectively (Table 1).

**Table 1:** Participant characteristics*

Characteristics		First Year Students N = 760		Final Year Students N = 695	
		n	%	n	%
Sex	Female	558	73.8	522	71.9
	Male	202	26.2	173	28.1
Fields of study	Medical	445	54.4	457	60.7
	Nursing	142	15.0	60	9.5
	Midwifery	50	2.3	59	2.3
	Other studies^**^	123	28.2	119	27.5
Nationality	Argentinean	658	88.2	662	95.4
	Foreign	102	11.8	33	4.6
Age/years	18–24	620	81.3	90	15.6
	25–29	94	12.8	493	69.3
	> 30	46	5.9	112	15.2
Marital status	Single	719	95.8	571	82.9
	Civil union/partnership	12	1.3	79	10.9
	Married	27	3.0	38	5.4
Children	None	708	95.1	648	94.1
Living with	Parental family	592	79.0	417	60.4
	Partner/children	63	7.19	149	20.8
	Alone	55	7.1	86	13.0
	Friends	33	4.7	19	3.3
Labour situation	Not working	451	60.2	374	53.3
	Part-time employee	184	25.1	211	30.2
	Independent worker	57	7.3	60	9.0
	Full-time employee	49	6.8	32	4.6
Father’s educational level^†^	Low	226	30.2	198	28.8
	Middle	330	43.7	277	41.2
	High	190	25.9	206	29.7
Mother’s educational level^†^	Low	203	27.5	149	21.2
	Middle	381	50.1	362	53.9
	High	162	22.3	175	24.7
Sexual debut	Yes	609	82.4	661	97.3
Use of condom in sexual debut	Yes	527	86.9	604	91.5
Current use of modern contraception	Yes	533	89.9	604	92.4
HIV testing	Yes	244	33.0	480	68.9

*Missing data not shown **Includes: nutrition (10.2%), radiology (7.7%), physiotherapy (7.2%), and speech therapy (2.4%) †Low: Illiterate, incomplete or complete primary school, incomplete high school; Middle: High school degree, complete or incomplete tertiary education, incomplete university; High: University degree

### Knowledge on current abortion law

**Figure 1:**
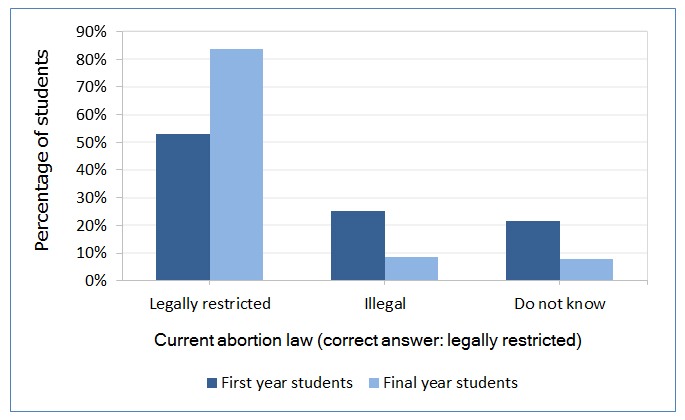
Healthcare students’ knowledge on current abortion law (first year N=732, final year N=679)

In responses to the question assessing knowledge of the current abortion law, we observed a significantly higher percentage of ‘legally restricted’ answers (which was the correct option), and a significantly lower percentage of ‘illegal’ and ‘do not know’ answers (p < 0.001) in final-year students than first-year students (Fig. 1). The option ‘always legal’ was not considered due to the low number of students who selected it (first year 1.2%: n = 8; final year 0.5%: n = 4).

Among final-year students, knowledge significantly differed by field of study *(*p < 0.001). Almost all medicine (94.9%) and midwifery students (96.6%) answered correctly (´legally restricted´), whereas nursing (73.5%) and control group (nutrition, radiology, speech therapy, physiotherapy) students (61.7%) were less likely to choose the correct option and more likely to admit not knowing (12.4% and 21.8% vs. none of medical and midwifery students).

In the responses of final-year students from the control group, we observed that law knowledge (‘legally restricted’) was significantly associated with age (63.5% of the students in the group aged 18–24 years chose ‘legally restricted’; 66.1% in the group aged 25–29 years; 38.5% in the group aged 30 years or more; p< 0.001). In this group of students (first-year control group) law knowledge was also significantly associated with parents’ educational levels (mother’s educational level: 39.4% of the students whose mothers had low educational level chose ‘legally restricted’ vs. 67% in the high educational level; father’s educational level: 49.1% of the students whose fathers had low educational level chose ‘legally restricted’ vs. 66.6% in the high educational level; p< 0.001).

**Figure 2:**
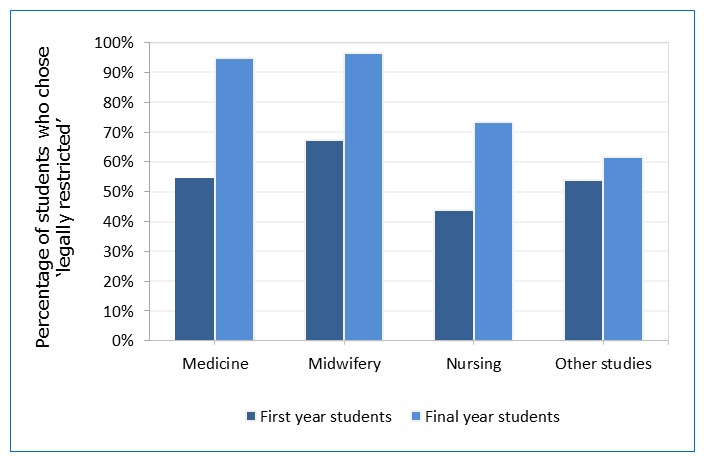
Percentage of healthcare students who chose the correct answer, by field of study (first year N=732, final year N=679)

We compared first and final-year students’ answers to the question on current abortion law. There was a significant increase in correct answers (´legally restricted´) in all fields of study, except for the control group (p< 0.001). We also observed a significant decrease in incorrect answers in medicine and midwifery students (p<0.001, p < 0.05 respectively; Fig. 2).

The 592 final-year students who responded correctly (´legally restricted´) were asked to identify the applicable circumstances from a list. The most commonly chosen were *woman’s life at risk* (87.4%), *rape of a woman with develop*

**Table 2:** Comparison of first- and final-year healthcare students’ knowledge of the circumstances in which access to abortion is legal in Argentina

Circumstances to access abortion			Legal					Not considered legal by current law	
			Life risk^*^	Rape of woman with developmental disability^*^	Rape	Health risk^*^	Mental health risk	Severe foetal malformations	Any foetal malformation^*^
Medicine	First year N = 234	n	192	153	141	55	46	88	9
		%	82.10	65.40	60.30	23.50	19.70	37.60	3.80
	Final year N = 423	n	384	375	283	112	57	250	2
		%	90.90	88.60	67.10	26.50	13.40	59.50	0.50
	p value		<0.05	<0.001			<0.05	<0.001	<0.05
Nursing	First year N = 59	n	53	43	42	23	15	22	4
		%	89.80	72.90	71.20	39	25.40	37.30	6.80
	Final year N = 42	n	34	34	33	7	9	20	5
		%	79.90	80.80	79.00	16.90	21.40	47.50	12.30
	p value					<0.05			
Midwifery	First year N = 33	n	22	20	26	4	8	9	0
		%	66.70	60.60	78.80	12.10	24.20	27.30	0.00
	Final year N = 56	n	46	44	42	15	8	38	1
		%	82.10	78.60	75.00	26.80	14.30	67.90	1.80
	p value							<0.001	
Other Studies	First year N = 65	n	45	42	39	16	10	24	3
		%	70	65.50	61.20	26	16.30	37.60	5.30
	Final year N = 71	n	55	55	48	29	13	36	5
		%	79.60	77.20	65.10	42.30	18.80	53.10	7.40
	p value					<0.05		<0.05	
TOTAL	First year N = 391	n	312	258	248	98	79	143	16
		%	79.10	66.20	62.40	25.80	19.50	37.30	4.50
	Final year N= 592	n	519	508	406	163	87	344	13
		%	87.40	85.40	67.90	29.00	15.20	57.40	2.90
	p value		<0.001	<0.001				<0.001	

*Circumstances in which there is a significant difference among final-year students by fields of study (p < 0.05)

*mental disabilities *(85.4%), and *rape* (67.9%). There were two other feasible options-*risk of woman’s health* and *risk of mental health-*chosen by only 29% and 15.7% respectively. The 57.4% of final-year students incorrectly identified *severe foetal malformations* as a legal circumstance (Table 2).

Moreover, we observed significant differences among final-year students from the different fields of study in the correct options. *Woman´s life risk *and *rape of a woman with developmental disabilities were *selected by almost 90% of medical students and around 80% of midwifery, nursing, and other studies’ students. *Health risk* was chosen by 42.3% of other, 26.5% of medical, 26.8% of midwifery, and 16.9% of nursing students (p< 0.05; Table 2).

Foreign nursing students of final year were less likely to identify *rape of a woman with developmental disabilities *as a cause for legal abortion (50% vs. 86.1% Argentinean nursing students; p* < *0.05). Among the control group students of final-year, the group aged 25-29 years was less likely to choose *woman’s health risk *and *risk for mental health* than younger and older students (group aged 18–24: 53.9% chose *woman’s health risk* and 30.4%* risk for mental health*; group aged 25–29: 30.9% and 10.9% respectively; group aged 30 or more: 56.7% and 23.6% respectively; p< 0.05).

Comparing final-year to first-year students, we observed a significant increase in the identification of some circumstances: *woman’s life risk* (79.1% first-year vs. 87.4% final-year), *rape of a woman with developmental disabilities* (66.2% vs. 85.4% last) and *severe foetal malformations* (37.3% vs. 57.4%), even though the latter is illegal (p< 0.001). We also compared the responses between first and final-year students according to their field of study (Table 2).

### Opinion on what the abortion law should be in Argentina

**Figure 3:**
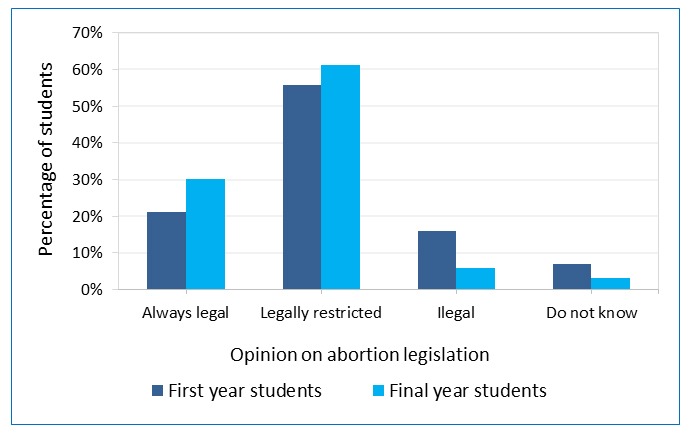
Healthcare students’ opinion on abortion legislation (first year N=705, final year N=676)

Students were asked if they thought that abortion should be ‘legal’, ‘legally restricted’, or ‘illegal’, or if they ‘did not know’ (Fig. 3). Personal opinions were provided by 705 first-year and 676 final-year students.

More final-year students thought that abortion should be legal or legally restricted compared to first year students (p < 0.001). When we analysed by field of study, this significant difference was observed in medicine, midwifery, and other studies (but not nursing).

Final-year students who were not currently using contraceptives were more likely to think that abortion should be legal (46.5% of the students not using contraceptives chose ‘legal’ vs. 29% of the students using contraceptive methods; p < 0.05). The students using contraceptives were more likely to choose ‘legally restricted’ (63.8% of the students using chose ‘legally restricted’ vs. 44.6% not using; p < 0.05).

The 387 first-year students and 411 final-year students who answered that abortion should be ‘legally restricted’ were asked in which circumstances they thought it should be permitted (Table 3). In final-year students, significant differences by field of study were observed. The options *severe foetal malformations* and *life risk* were chosen by almost all medical and midwifery students; fewer nursing and other students chose them (p < 0.05). Medical and midwifery students were also more likely to select *woman’s request in fewer than 12 weeks of pregnancy *and *failure of *

**Table 3:** First and final-year students’ personal opinions on when abortion should be legal

Circumstances in which abortion should be legal			Legal by current law					Not considered legal by current law				
			Rape	Life risk*	Rape of a woman with developmental disabilities	Health risk	Mental health risk	Severe foetal malformations*	Woman´s will less than 12 weeks of pregnancy*	Any foetal malformation*	Lack financial resources	Contraceptive failure*
Medicine	First year N = 219	n	193	158	159	90	60	136	8	18	19	13
		%	88.10	72.10	72.60%	41.10	27.40	62.10	3.70	8.20	8.70	5.90
	Last year N = 263	n	248	255	240	147	121	251	57	34	33	28
		%	94.10	96.90	91.30	55.80	45.90	95.50	21.20	13.00	12.50	10.70
p value			0.05	0.001	0.001	0.05	0.001	0.001	0.001			
Nursing	First year N = 65	n	59	43	51	32	20	36	5	18	8	4
		%	90.80	66.20	78.50	49.20	30.80	55.40	7.70	27.70	12.30	6.10
	Last year N = 37	n	35	31	30	16	15	32	3	14	2	0
		%	94.80	83.80	81.70	42.90	40.30	86.40	7.80	38.20	5.20	0.00
p value								0.05				
Midwifery	First year N = 30	n	28	22	26	13	7	17	2	2	1	1
		%	93.30	73.30	86.70	43.30	23.30	56.70	6.70	6.70	3.30	3.30
	Last year N = 36	n	34	33	29	20	19	35	7	8	4	3
		%	94.40	91.70	80.60	55.60	52.80	97.20	19.40	22.20	11.10	8.30
p value							0.05	0.05		0.05		
Other studies	First year N = 73	n	71	49	62	46	31	46	2	20	6	4
		%	97.80	80.40	84.70	63.10	42.50	65.30	3.10	29.50	8.00	5.80
	Last year N = 75	n	70	61	64	40	30	64	8	9	7	3
		%	92.30	82.70	84.70	54.10	42.60	85.20	10.90	11.80	9.40	4.20
p value								0.001	0.05	0.05		
Total students	First year N = 387	n	351	282	298	181	118	235	17	58	34	22
		%	91.80	74.10	77.70	49.40	32.70	62.10	4.10	17.70	8.80	5.90
	Last year N = 411	n	387	380	363	223	185	382	75	65	46	34
		%	93.70	91.40	88.20	54.00	44.60	91.70	16.80	15.30	10.90	7.70
p value				0.001	0.001		0.001	0.001	0.001			

*Circumstances in which there was a significant difference among final-year students by fields of study (p < 0.05)

*contraceptive method* (p < 0.05). Nursing students were more likely to select *any foetal malformation *than the rest (p <0.001), particularly foreign nursing students (67.4% of foreign nursing students chose any foetal malformation vs. 29% of Argentinean nursing students; p < 0.05; Table 3).

#### Opinion on what they have learned

Out of 393 final-year students, 89.8% of midwifery, 78.7% of nursing, and 61.6% of medicine students completely agreed that they had learned about sexual and reproductive rights at the university, in contrast to only 24.1% of the control students (p < 0.001).

## Discussion

In our previous study, we identified that half of the first-year healthcare students from the School of Medicine of the UBA failed to identify the current legal regulations on abortion; the ones who knew about the law could not identify the circumstances in which abortion was permitted. In the present study, we assessed final-year students´ knowledge of the abortion law and inferred what they learned throughout their medical courses.

Since healthcare professional programs last six years, it would have taken many years to survey the same cohort of students; consequently, we decided to administer our questionnaire to first- and final-year students during the same academic year. We were urged to perform this investigation in order to encourage changes in the curricula, which is currently under revision. Furthermore, abortion is on the public agenda; therefore, the longer it would take us to reach a conclusion, the more likely would it be for students´ knowledge to be influenced by the social context. Thus, it would have been difficult to assess the medical curriculum’s influence on students´ knowledge and opinion, separately from the effects of the environment.

A larger majority (83.7%) of final-year students but only 53.1% of the first-year students knew about the current abortion law (Fig. 1). This difference was significant in medical, midwifery, and nursing students (not in the control group) (Fig. 2). Final-year medical (94.9% correct answers), midwifery (96.6%), and nursing (73.5%) students had higher knowledge of current legislation than first-year students (53.1%) and the control group, who do not receive sexual and reproductive health education (61.8%).

However, when students were asked to provide more detailed information and to identify the circumstances in which abortion was legal, the results were not encouraging.

We believe that healthcare providers who know when an abortion is legal are more likely to provide sound information and ensure it is appropriately performed. Increased knowledge and improved attitudes among healthcare providers have the potential to reduce barriers to legal and safe abortion care by reducing stigma and reluctance to provide abortion.^25^

Final-year students were likely to identify only three circumstances more frequently than first-year students were: *woman’s life risk* (final-year 87.4% vs. first-year 79.1%), *rape of a woman with developmental disabilities* (final-year 85.4% vs. first-year 66.2%), and the incorrect option *severe foetal malformations *(final-year 57.4% vs. first-year 37.3%; Table 2).

However, in the analysis by field of study, final-year midwifery and nursing students did not show higher identification of current legal circumstances compared to first-year students. Actually, a decrease in the identification of *woman’s health risk* (first-year 39% vs. final-year 16.9%) was observed among nursing students. Like in the control group, first-year midwifery and nursing students appeared to have the same knowledge as final-year students. It could be inferred that midwifery and nursing students do not receive substantial information about abortion in their curricula.

On the other hand, a higher percentage of final-year medical students selected *woman’s life risk* (first-year 82.1% vs. final-year 90.9%) and *rape of a woman* *with developmental disabilities* (first-year 65.4% vs. final-year 88.6%). In this case, we could infer that the information medical students receive is restricted to certain circumstances. We even observed a decrease in the selection of *woman’s mental health risk* by final-year medical students in comparison with first-year medical students (19.7% first year vs. 13.4% final-year).

It is not clear whether university curriculums are deliberately designed to avoid covering the legal aspects of abortion or whether the faculty lack knowledge of this topic. Research from another country where abortion is legally restricted showed that many doctors are uncertain about the legality of abortion in their regions, including those actually practicing abortion.^24^

Unexpectedly, we found that final-year control group students were significantly more likely than all other students to choose *woman’s health risk* (42.3% final-year control students vs. 29% average final-year). We observed no differences in the option *rape* by fields of study or academic year. In first-year students, there was almost no difference between this option and *rape of a woman with developmental disabilities*. Final-year medical students were more likely to choose the latter. There was greater misidentification of *severe foetal malformations* in final-year than first-year students (Table 2). This was consistent with the difference observed when students were asked about their personal opinion regarding the circumstances in which abortion should be legal (first year 62.1% vs. final year 91.7%). This belief was observed in other surveys of medical students; in one such study, 72.8% of the students agreed that it was acceptable for women to choose abortion because of foetal anomaly or congenital disorder.^22^ Their opinion that foetal malformation should be a legal justification may have influenced them to think that it is actually legal. Conversely, we did not observe a significant relationship between knowledge and* rape*, although most students´ personal opinions were that it should be a legal justification (93.7%).

Overall, 91.1% of final-year students were pro-abortion (answered that abortion should be legal and legally restricted). This result is higher than the one observed in our first-year student survey (Fig. 3)^16^. Only 30.1% of final-year students responded that abortion should always be legal, compared to percentages of 40%^23^ and 70%^22^ reported in surveys of medical students in other countries. Final-year students were more likely to favour legal abortion and fewer thought abortion should be illegal.

A limitation of our study is that it did not take into consideration whether the students had participated in courses outside the university where they might have learned about the current abortion legislation.

## Conclusions

Although most final-year healthcare students knew that abortion is legally restricted in Argentina, we found misconceptions regarding the circumstances under which abortion is legal among students from all fields of study (medical, midwifery, nursing and other studies). This demonstrates that abortion is still inadequately addressed in the medical school curricula.

Previous studies have shown that education about abortion is acceptable and valued by healthcare students and should be integrated into the curricula of all health professions. Nurses and midwives should have a relevant role in expanding access to abortion services, as is the situation in many countries.^21^^, ^^22^^, ^^26^^, ^^27^

Medical schools should ensure that their students understand the laws related to and responsibilities encompassing their future professional practice regarding abortion care, regardless of their personal opinion. Medical schools should also ensure that sexual and reproductive health is an integral component of their medical school curricula. Healthcare providers who know circumstances under which an abortion is legal are more likely to provide sound information and ensure it is performed appropriately.

### Acknowledgements

Financial support of the Letten Foundation and from the grant program university-extension UBANEX University of Buenos Aires (UBA) is noted.

We thank Soledad García Conde and Martin Romeo for their collaboration with the statistical analysis, and Leticia Azzaretti, Marina Ridao, Silvia Chera, and Carlota Ramirez for their help throughout different portions of the project. Students of the Facultad de Medicina, UBA and from the University of Oslo collaborated in the process of data collection. UBA: Juan P, Bessia, Natividad Burdisso, Paola Carabajal, Matías Calos, Adriana Diaz Balocchi, Guillermina Dimilito, Carolina Fernandez, Mariano Granero, Vanesa Guerrieri, Florencia Hershson, Estefania Kuzmicki, Justina Longo, Natalia Mandel, Melanie Mysler, Noelia Saavedra, Margarita Satostegui, Agustina Svoboda; UIO: Andrea Brodahl, Magrit Hovind, Magnus Nakkim, Nicolas Fernandez, Linda Hestvik, Harald Hovik.

### Conflict of Interest

The authors declare that they have no conflict of interest.
